# Supplementary material for: Descending inhibitory rostral ventromedial medulla neurons cause widespread antinociception and contribute to the pain-inhibits-pain phenomenon
Source: Nat Commun. 2026 Apr 2;17:4765. doi: 10.1038/s41467-026-71289-z (PMC13216533; doi:10.1038/s41467-026-71289-z)
Supplement: Supplementary file 1 — Supplementary Information [file 41467_2026_71289_MOESM1_ESM.pdf]

Descending Inhibitory Neurons of the RVM Cause Widespread Bilateral Antinociception and Contribute to the Pain-Inhibits-Pain Phenomenon

Robert P. Ganley, Marília Sousa, Guangchen Ji, Matteo Ranucci, Camilla Beccarini, Kira Werder, Francesca Pietrafesa, Simon d'Aquin, Tugce Akyüz, Michèle Hubli, Petra Schweinhardt, Volker Neugebauer, Mark A. Hoon, Hendrik Wildner & Hanns Ulrich Zeilhofer

Supplementary figures

Supplementary Fig. 1. Intersectional targeting of medial RVM descending inhibitory neurons (related to Fig. 1).

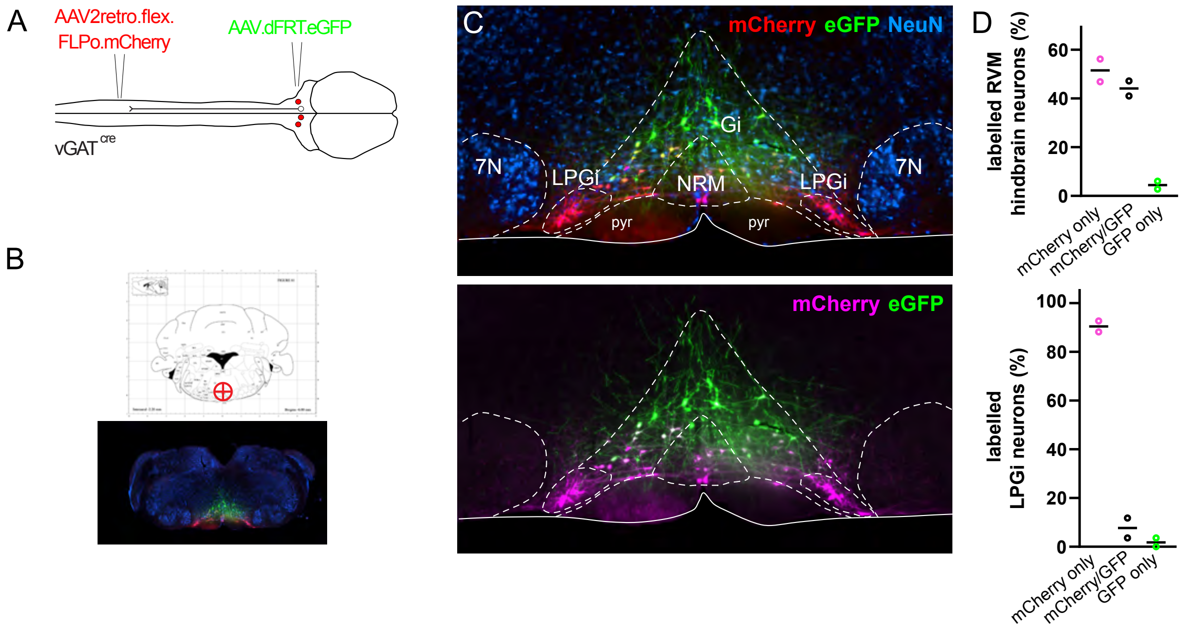

A. Injection scheme for the intersectional labeling of vGAT RVM^{SC}neurons, **B**. Injection coordinates used in preliminary experiments to target descending inhibitory neurons and example injection site from a vGAT^{cre} animal that received these injections. **C**. Larger annotated image illustrating the location of eGFP- and mCherry-labeled neurons. Note the many neurons that only contain mCherry are found in the LPGi, whereas eGFP-labeled neurons are only present around the midline. **D**. Quantification of RVM neurons that are labeled with mCherry and/or eGFP, showing around 44.1% of cells contain both mCherry and eGFP (n = 2 female mice). Below, quantification of labeled neurons within the LPGi that contain mCherry, and/or eGFP indicating only 7.7% of these cells contain mCherry and eGFP. Scale bar, 200 μm. 7N, facial nucleus; LPGi, lateral paragigantocelular nucleus; NRM, nucleus raphe magnus; Gi, gigantocellular reticular nucleus; pyr, pyramidal tract.

Supplementary Fig. 2: hM3Dq expression is required for changes in sensitivity mediated by CNO (related to Fig. 3)

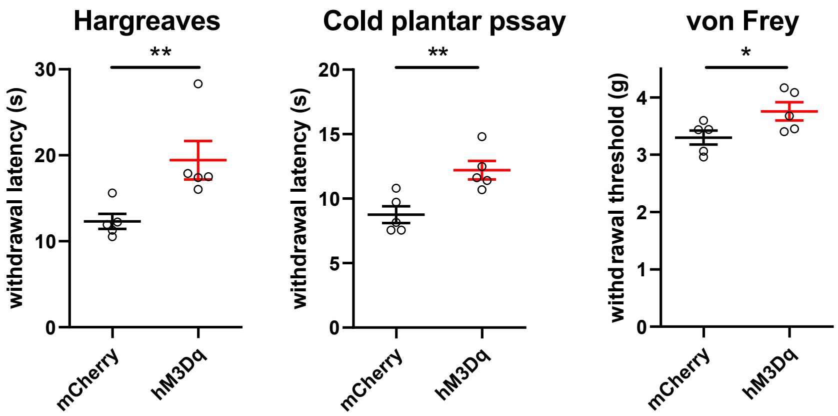

Withdrawal latencies are increased in Hargreaves and cold plantar assays in hM3Dq-expressing animals following CNO injection compared to mCherry-expressing controls (Hargreaves Mann Whitney test: p= 0.0079, Cold Plantar Assay Unpaired t-test: p = 0.0072). Withdrawal thresholds to electronic von Frey stimulation were increased in hM3Dq-expressing animals when compared to mCherry-expressing controls (unpaired t-test p = 0.0268). All experiment were done in female mice. All data are shown with mean ± SEM. hM3Dq: n = 5 mice, mCherry n = 5 mice. *, $p < 0.05$; **, $p < 0.01$.

Supplementary Fig. 3: Effects of selective chemogenetic and optogenetic stimulation of the spinal vGAT RVM^{SC} neuron terminals on nociceptive responses (A-F), and of optogenetic stimulation on noxious stimulus induced activity of dorsal horn WDR neurons (G,H) (related to Fig. 3)

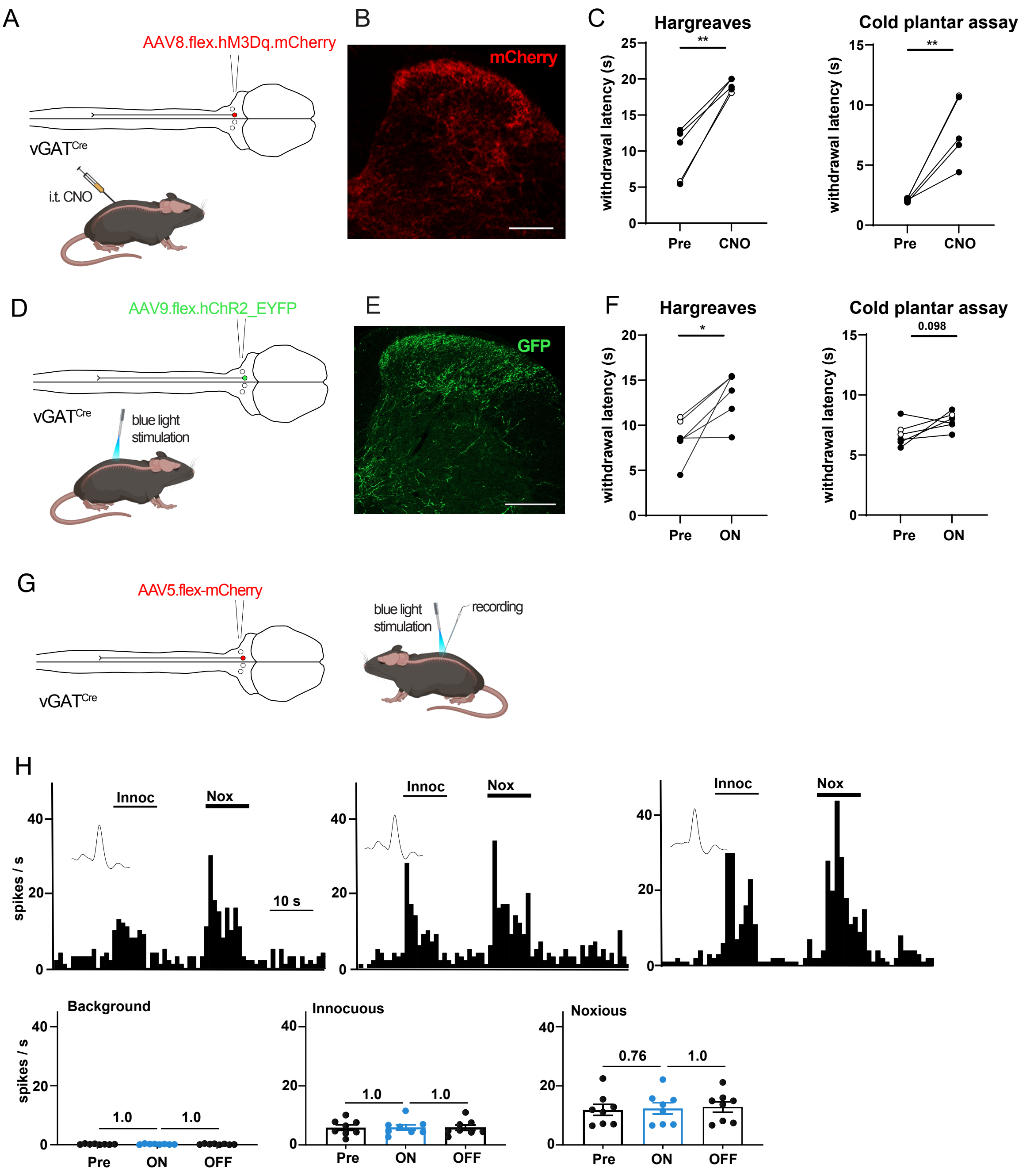

A-C. Selective chemogenetic stimulation of the spinal vGAT RVM^{SC} neuron terminals. **A.** injection scheme for the chemogenetic activation of the spinal cord terminals of vGAT^{Cre} RVM neurons with intrathecal CNO delivery (1 μg in 10 μl). **B.** Transverse dorsal horn section with axon terminals labelled in the superficial dorsal horn of the lumbar spinal cord (scale bar, 200 μm). **C**. Behavioral tests (intrathecal injection of CNO, n = 5 mice) (paired t-tests: Hargreaves: p = 0.002; cold plantar assay: p = 0.008). **D-F.** Same as A-C, but optogenetic stimulation (0.2 ± 0.06 mW [1.72 mW / mm^2] applied at 20 Hz for 1 s, every 10 s) (paired t-tests Hargreaves: p = 0.002; cold plantar assay: p = 0.098, n = 6 mice). **G,H.** Extracellular single-unit recordings of WDR dorsal horn neurons in vGAT^{cre} mice injected

with a control vector (rAAV5.flex.mcherry). **G.** Experimental design. **H.** Top row: peristimulus time histograms show action potentials (spikes / s) evoked by innocuous (Innoc) and noxious (Nox) mechanical stimulation of one hindpaw in an individual WDR neuron before (pre blue-light), during (blue-light on, 2 min) and after (blue-light off, 2 min) stimulation. Insets show individual action potential traces. Bottom row: summary of effects. Blue light had no effects on background (n = 8), and innocuous (n = 8) and noxious (n = 8) mechanical stimulation evoked neuronal activity (one-way repeated measure ANOVA, $F(1.7,11.6) = 0.079$, $p = 0.89$, n = 8; and $F(1.6,11.1) = 1.6$, $p = 0.25$, n = 8, for innocuous and noxious stimulation, respectively). Numbers of above horizontal bars are p values for pairwise post-hoc comparisons. Bar histograms show mean ± SEM for the sample of neurons. Open and closed symbols indicate female and male mice, respectively.

Supplementary Fig. 4: vGAT RVM^{SC} inhibitory neurons use both GABA and glycine as neurotransmitters (related to Fig. 4)

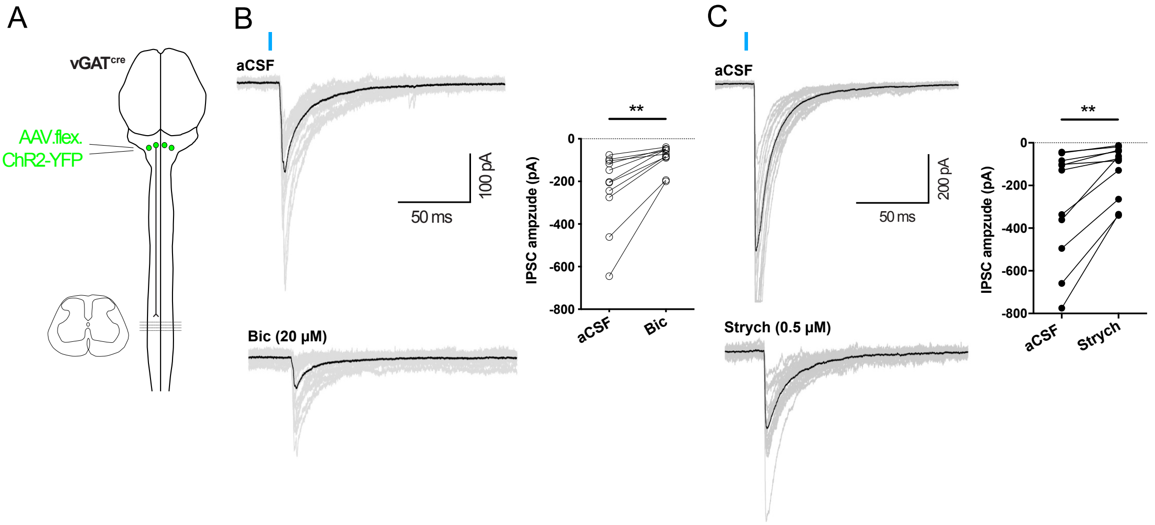

A. Brain injection scheme for optogenetic activation of vGAT axon terminals in spinal cord slices. **B.** Light-evoked IPSCs recorded from dorsal horn neurons before and after bath application of bicuculline. Average traces are shown in black and are composed of 10 sweeps in each condition, which are shown in grey. Right: group data of the amplitude of light-evoked IPSCs (4 ms, 470 nm light) before and after bicuculline application, showing a significant reduction in current amplitude (Wilcoxon matched-pairs signed rank test: $p = 0.0010$, n = 11 cells). **C.** Example of light-evoked IPSCs that are reduced in amplitude by strychnine application, average traces are black and consists of 10 individual sweeps shown in grey. Blue lines indicate the time of blue-light stimulation. Right: group data showing that light-evoked IPSC amplitudes are significantly reduced by strychnine application (Wilcoxon matched-pairs signed rank test: $p = 0.0010$, n = 11). **, $p < 0.01$. Open and closed symbols indicate female and male mice, respectively.

Supplementary Fig. 5: Chemogenetic activation of descending vGAT RVM^{SC} neurons reduces heat and cold sensitivity of the contralateral paw (related to Fig. 4)

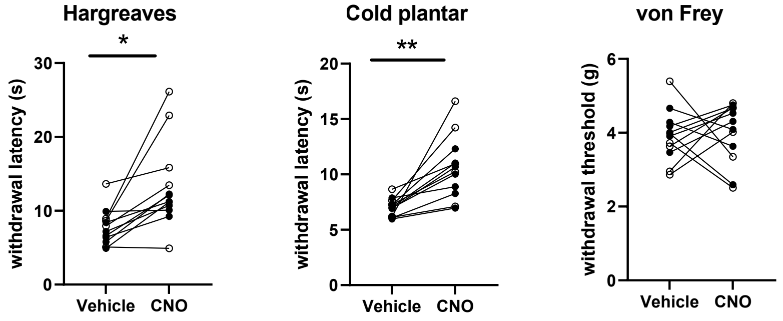

Withdrawal latencies on the contralateral paw were increased in the thermal Hargreaves plantar assay and in the cold plantar assay following CNO injection relative to vehicle injected controls (paired t-test Hargreaves p = 0.0038, cold plantar assay p = 0.0008). Withdrawal thresholds to the electronic von Frey were not altered in the contralateral paw following chemogenetic activation of these neurons (paired t test p = 0.84). n = 12 mice, significance levels; *, p < 0.05; **, p < 0.01; ***, p < 0.001. Open and closed symbols indicate female and male mice, respectively.

Supplementary Fig. 6: Chemogenetic activation of descending vGAT RVM^{sc} neurons reverses inflammation-induced hypersensitivity in the contralateral paw (related to Fig. 6)

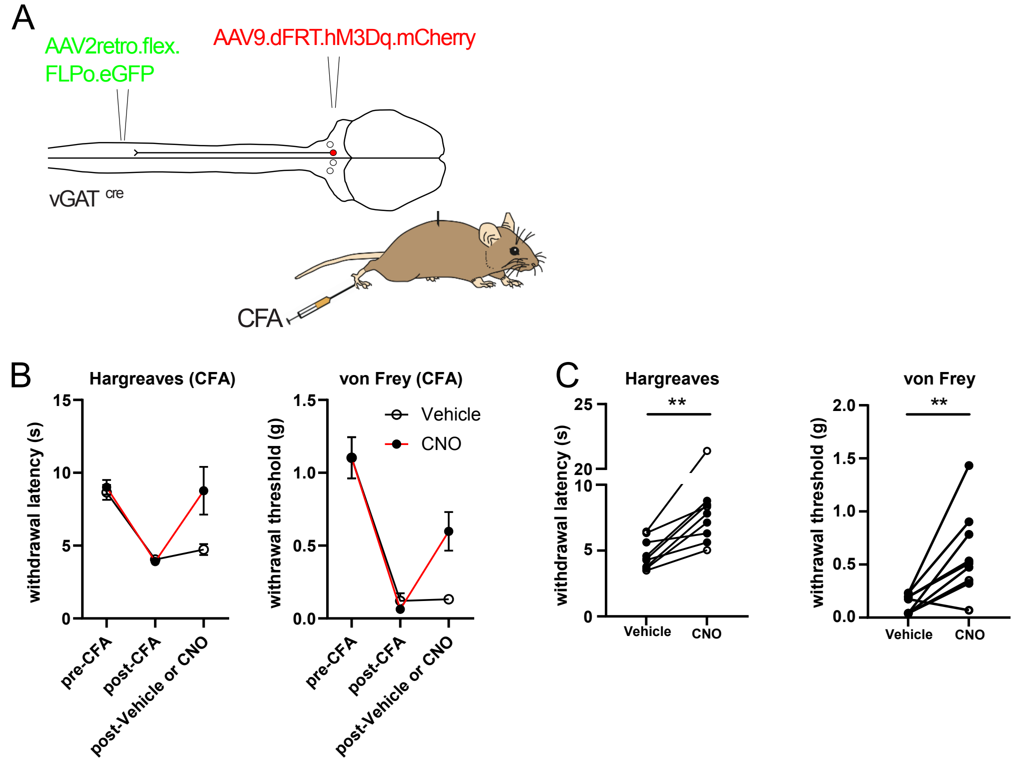

A. Experimental design for activation of descending vGAT RVM^{SC} neurons retrogradely labeled from the left lumbar dorsal horn, and to induce inflammatory hyperalgesia in the right paw. **B.** Withdrawal latencies to thermal stimuli and withdrawal thresholds to mechanical stimuli on the contralateral paw were decreased by intraplantar injection of CFA, which was reversed by chemogenetic activation of vGAT RVM^{SC} neurons (n = 9 mice) (two-way repeated measures ANOVA, significant interaction of treatment x time Hargreaves (F(2,32) =

4.87, $p = 0.020$; Mixed-effects analysis, significant interaction of treatment x time von Frey $F(2, 32) = 4.78$, $p = 0.015$). **C.** Withdrawal thresholds and latencies for each animal following CFA-induced hypersensitivity and after i.p. CNO or vehicle injection (Wilcoxon matched-pairs signed rank tests: Hargreaves p= 0.0078, von Frey p = 0.0078). Significance levels; ** $p < 0.01$. Open and closed symbols in panel C indicate female and male mice, respectively.

Supplementary table 1: Antibodies used in immunostaining experiments

Antibody (dilution)	Supplier/Source	Cat#/RRID
Chicken anti-GFP (1:1000)	LifeTech	A10262/AB_2619988
Goat anti-mCherry (1:500)	Sicgen	AB0081-200/AB_2333094
Rabbit anti-mCherry (1:500)	Thermo Fisher Scientific	PA5-34974/AB_2552323
Guinea pig anti-NeuN (1:1000)	Synaptic systems	266004/AB_2619988
Rabbit anti-c-Fos (1:500)	Calbiochem	PC38/AB_2106755
Rabbit anti-c-Fos (1:1000)	Cell Signalling	2250/ AB_2247211
Rabbit anti-vGAT (1:1000)	Synaptic Systems	131003/AB_887869
Guinea pig anti-vGAT (1:1000)	Thermo Fisher Scientific	PA5-111832/AB_2857241
Alexa 488-Donkey anti-Chicken (1:500)	Jackson ImmunoResearch	703-546-155/AB_2340376
Cy3-Donkey anti-goat (1:500)	Jackson ImmunoResearch	705-166-147/AB_2340413
Alexa 488-Donkey anti-goat (1:500)	Jackson ImmunoResearch	705-545-003/AB_2340428
Cy5-Donkey anti-goat (1:500)	Jackson ImmunoResearch	705-175-147/AB_2340415
Alexa 488-Donkey anti-guinea pig (1:500)	Jackson ImmunoResearch	706-545-148/AB_2340472
Alexa 647-Donkey anti-guinea pig (1:500)	Jackson ImmunoResearch	706-605-148/AB_2340476
Cy5-Donkey anti-guinea pig (1:500)	Jackson ImmunoResearch	706-175-148/AB_2340462
Alexa 488-Donkey anti-rabbit (1:500)	Jackson ImmunoResearch	711-545-152/AB_2313584
Alexa 647-Donkey anti-rabbit (1:500)	Jackson ImmunoResearch	711-607-003/AB_2340626
Cy3-Donkey anti-rabbit (1:500)	Jackson ImmunoResearch	711-165-152/AB_2307443

Supplementary table 2: RNA probes used in multiplex fISH experiments

mRNA target	Supplier/Source	Cat#
EGFP	Advanced Cell Diagnostics	400281
EGFP-C3	Advanced Cell Diagnostics	400281-C3
Mm-Slc17a6	Advanced Cell Diagnostics	319171
Mm-Slc17a6-C2	Advanced Cell Diagnostics	319171-C2
Mm-Slc17a6-C3	Advanced Cell Diagnostics	319171-C3
Mm-Slc32a1	Advanced Cell Diagnostics	319191
Mm-Slc32a1-C2	Advanced Cell Diagnostics	319191-C2
tdTomato	Advanced Cell Diagnostics	317041
3-plex negative control probe	Advanced Cell Diagnostics	320871
RNAscope Fluorescent Multiplex Reagent Kit	Advanced Cell Diagnostics	320850
RNAscope Fluorescent Multiplex Reagent Kit v2	Advanced Cell Diagnostics	323100

Supplementary table 3: AAV vectors used in study

virus name	Full name	Supplier/Source	Cat#
AAV2retro.eGFP	ssAAV-retro/2-CAG-EGFP-WPRE-SV40p(A)	Viral Vector facility UZH/ETHZ	V24-retro
AAV2retro.flex.eGFP	ssAAV-retro/2-shortCAG-dlox-EGFP(rev)-dlox-WPRE-SV40p(A)	Viral Vector facility UZH/ETHZ	V158-retro
AAV2retro.flex.mCherry	ssAAV-retro/2-shortCAG-dlox-mCherry(rev)-dlox-WPRE-hGHp(A)	Viral Vector facility UZH/ETHZ	V116-retro
AAV2retro.flex.FLPo.BFP	ssAAV-retro/2-hSyn1-chI-dlox-EBFP2_2A_FLPo(rev)-dlox-WPRE-SV40p(A)	Viral Vector facility UZH/ETHZ	V175-retro
AAV2retro.flex.FLPo.mCherry	ssAAV-retro/2-hSyn1-chI-mCherry_2°_FLPo-WPRE-SV40p(A)	Viral Vector facility UZH/ETHZ	V173-retro
AAV2retro.flex.FLPo.eGFP	ssAAV-retro/2-hSyn1-chI-EGFP2_2A_FLPo-WPRE-SV40p(A)	Viral Vector facility UZH/ETHZ	V171-retro
AAV2retro.flex.FLPo	ssAAV-retro/2-pEF1a-FLPo-WPRE-hGHpA	Addgene	87306-AAVrg
AAV9.flex.ChR2-YFP	ssAAV-9/2-hEF1a-dlox-hChR2(H134R)_EYFP(rev)-dlox-WPRE-hGHp(A)	Viral Vector facility UZH/ETHZ	v214-9
AAV8.FRT.tdTomato	ssAAV-8/2-hSyn1-dlox-tdTomato(rev)-dlox-WPRE-bGHp(A)	Viral Vector facility UZH/ETHZ	v284-8
AAV9.FRT.eGFP	ssAAV-9/2-hSyn1-chI-dFRT-EGFP(rev)-dFRT-WPRE-hGHp(A)	Viral Vector facility UZH/ETHZ	V335-9
AAV8.FRT.hM3D(q).mCherry	ssAAV-8-hSyn1-dFRT-hM3D(Gq)-mCherry	Viral Vector facility UZH/ETHZ	V189-8
AAV9.FRT.hM3D(q).mCherry	ssAAV-9-hSyn1-dFRT-hM3D(Gq)-mCherry	Viral Vector facility UZH/ETHZ	V189-9
AAV9.FRT.ChR2-YFP	ssAAV-2-hSyn1-chI-dFRT-hChR2(H134R)_EYFP(rev)-dFRT-WPRE-hGHp(A)	Viral Vector facility UZH/ETHZ	V331-9
AAV9.FRT.eGFP.TeTxLC	ssAAV-9/2-hSyn1.chl-dFRT-EGFP-2A-FLAG:TeTxLC(rev)-dFRT-WPRE-hGHp(A)	Viral Vector facility UZH/ETHZ	v450-9
AAV9.hEF1a.DIO.ChR2	ssAAV-9-hEF1α-dlox-hChR2(H134R)_EYFP(rev)-dlox-WPRE-hGHp(A)	Viral Vector facility UZH/ETHZ	v214-9
rAAV5.EF1a.DIO.mcherry		UNC	AV4311G

Supplementary table 4: Chemicals/Drugs used in study

Reagent	Source	Identifier
Clozapine-N-oxide	Enzo life sciences	BBL-NS105-0025
Bicuculline-methochloride	Alomone labs	B-137
Strychnine hydrochloride	Sigma Aldrich	S8753-25G
40% acrylamide solution	Thermofisher Scientific	HC2040
Histodenz™	Sigma Aldrich	D2158
4-hydroxytamoxifen	Tocris	3412
Capsaicin	Sigma Aldrich	M2028
Avidin, Alexa Fluor™ 488 conjugate	Thermofisher Scientific	A21370

Supplementary table 5: Mouse lines used in study

Mouse line	Supplier/source	Reference
vGAT^{cre} (all experiments except *in vivo* electrophyiological recordings)	Jackson lab	#016962/ IMSR_JAX:016962
vGAT^{cre} (*in vivo* electrophyiological recordings)	Jackson lab	#028862; B6J.129S6(FVB)-Slc32a1tm2(cre)Lowl/MwarJ
TRAP2	Jackson lab	#030323/ IMSR_JAX:030323
Ai65	Jackson lab	#021875/ IMSR_JAX:021875

Supplementary table 6: Software used in study

Software	Supplier/source	Identifier
GraphPad Prism version 8	GraphPad software	https://www.graphpad.com/scientific-software/prism/
Affinity Designer 2	Affinity serif	https://affinity.serif.com/en-gb/designer/
ImageJ FIJI	National Institutes of Health (NIH)	https://imagej.net/software/fiji/
Adobe Photoshop 2025	Adobe	https://www.adobe.com/home
Neurostar Stereodrive	Neurostar	https://robot-stereotaxic.com/motorized-stereotaxic-instrument/
Zen 2.3 Blue edition	Carl Zeiss	https://www.zeiss.com/microscopy/int/ downloads
HEKA Patchmaster v2.11	HEKA Elektronik	http://www.heka.com/downloads/ software/
Igor Pro 6.22A	Wavemetrics	https://www.wavemetrics.com/ downloads
MesoSPIM control	www.MesoSPIM.org	https://github.com/mesoSPIM/mesoSPIM-control
CellProfiler 2.2.0	www.cellprofiler.org	https://cellprofiler.org/previous-releases
