# Supplementary material for: Descending inhibitory rostral ventromedial medulla neurons cause widespread antinociception and contribute to the pain-inhibits-pain phenomenon
Source: Nat Commun. 2026 Apr 2;17:4765. doi: 10.1038/s41467-026-71289-z (PMC13216533; doi:10.1038/s41467-026-71289-z)
Supplement: Supplementary file 2 — Description of Additional Supplementary Files [file 41467_2026_71289_MOESM2_ESM.pdf]

Description of Additional Supplementary Files

Supplementary Video 1: descending vGAT RVM^{SC} neurons in a cleared brain.
Lightsheet microscope scan of vGAT RVM^{SC} neurons labelled with eGFP using an intersectional approach. Retrograde labeling was done from a single injection site (left lumbar dorsal horn). Note that axons emanate from the cell bodies in both a caudal and rostral direction. Caudal, up, rostral, down, scanning direction (video) is from the ventral to the dorsal surface (see inset)

Supplementary Video 2: Spontaneous flinching of mice following vGAT RVM^{SC} neuron silencing.
Mouse on the right (vGATc^{re}) displays spontaneous flinching of the left hindlimb throughout the video. Mouse on the right is a control mouse (Cre negative littermate) that received the same injections as the vGAT^{cre} mouse on the left.
