# Supplementary material for: Descending inhibitory rostral ventromedial medulla neurons cause widespread antinociception and contribute to the pain-inhibits-pain phenomenon
Source: Nat Commun. 2026 Apr 2;17:4765. doi: 10.1038/s41467-026-71289-z (PMC13216533; doi:10.1038/s41467-026-71289-z)
Supplement: Supplementary file 5 — Reporting Summary [file 41467_2026_71289_MOESM5_ESM.pdf]

nature portfolio

Corresponding author(s): Hanns Ulrich Zeilhofer

Last updated by author(s): 13/03/2026

Reporting Summary

Nature Portfolio wishes to improve the reproducibility of the work that we publish. This form provides structure for consistency and transparency in reporting. For further information on Nature Portfolio policies, see our Editorial Policies and the Editorial Policy Checklist.

Statistics

For all statistical analyses, confirm that the following items are present in the figure legend, table legend, main text, or Methods section.

n/a	Confirmed	
☐	☒	The exact sample size (*n*) for each experimental group/condition, given as a discrete number and unit of measurement
☐	☒	A statement on whether measurements were taken from distinct samples or whether the same sample was measured repeatedly
☐	☒	The statistical test(s) used AND whether they are one- or two-sided<br>*Only common tests should be described solely by name; describe more complex techniques in the Methods section.*
☐	☒	A description of all covariates tested
☐	☒	A description of any assumptions or corrections, such as tests of normality and adjustment for multiple comparisons
☐	☒	A full description of the statistical parameters including central tendency (e.g. means) or other basic estimates (e.g. regression coefficient) AND variation (e.g. standard deviation) or associated estimates of uncertainty (e.g. confidence intervals)
☐	☒	For null hypothesis testing, the test statistic (e.g. *F*, *t*, *r*) with confidence intervals, effect sizes, degrees of freedom and *P* value noted<br>*Give P values as exact values whenever suitable.*
☒	☐	For Bayesian analysis, information on the choice of priors and Markov chain Monte Carlo settings
☒	☐	For hierarchical and complex designs, identification of the appropriate level for tests and full reporting of outcomes
☒	☐	Estimates of effect sizes (e.g. Cohen's *d*, Pearson's *r*), indicating how they were calculated

Our web collection on statistics for biologists contains articles on many of the points above.

Software and code

Policy information about availability of computer code

Data collection	Zeiss Zen software: capturing confocal and epifluorescent microscope images<br>Spike2 software (version 4, Cambridge Electronics Design); used for spike sorting for in vivo recording experiments<br>Patchmaster software (v2x80): used to record whole-cell electrophysiology data
Data analysis	FIJI with cell counter plugin: manual quantification of imaging data<br>IGOR with NeuroMatic tools: analysis of electrophsiological data<br>Spike2 software (version 4: Cambridge Electronics Design)); analysis of in vivo action potential firing<br>GraphPad Prism 8: Statistical analysis of behavioural, anatomical, and electrophysiological data

For manuscripts utilizing custom algorithms or software that are central to the research but not yet described in published literature, software must be made available to editors and reviewers. We strongly encourage code deposition in a community repository (e.g. GitHub). See the Nature Portfolio guidelines for submitting code & software for further information.

Data

Policy information about availability of data

All manuscripts must include a data availability statement. This statement should provide the following information, where applicable:

- Accession codes, unique identifiers, or web links for publicly available datasets
- A description of any restrictions on data availability
- For clinical datasets or third party data, please ensure that the statement adheres to our policy

All analyzed data are included in the manuscript. Raw data will be uploaded to https://datadryad.org/stash upon publication of the manuscript

Research involving human participants, their data, or biological material

Policy information about studies with human participants or human data. See also policy information about sex, gender (identity/presentation), and sexual orientation and race, ethnicity and racism.

Reporting on sex and gender	N/A
Reporting on race, ethnicity, or other socially relevant groupings	N/A
Population characteristics	N/A
Recruitment	N/A
Ethics oversight	N/A

Note that full information on the approval of the study protocol must also be provided in the manuscript.

Field-specific reporting

Please select the one below that is the best fit for your research. If you are not sure, read the appropriate sections before making your selection.

☒ Life sciences ☐ Behavioural & social sciences ☐ Ecological, evolutionary & environmental sciences

For a reference copy of the document with all sections, see nature.com/documents/nr-reporting-summary-flat.pdf

Life sciences study design

All studies must disclose on these points even when the disclosure is negative.

Sample size	No sample sizes were determined prior to the study, but are similar to those reported in previous publications within the same research field using the same assays. Francois et al, 2017 Neuron 93: 822-839, Gu et al, 2023 Nature Neuroscience 26(4) 594-605
Data exclusions	Mice were excluded from behavioral experiments if injection sites were not successful, determined at the end of the experiment by histological techniques<br>Cells were excluded from electrophysiological analysis if access resistance changed >30% during the recording<br>in vivo units were only included if they responded to both innocuous (100g/6mm) and noxious (500g/6mm) mechanical stimulation
Replication	For all labeling and quantification experiments, (with the exception of fig8C), at least 3 independent animals were used and all gave similar results.<br>Results from repeated behavioral experiments are shown in all graphs and indicate the reproducibility of the effects in each assay
Randomization	The order for testing animals was randomised, but ensuring that roughly equal numbers were included for each treatment group/genotype
Blinding	For chemogenetic experiments, a second experimenter filled the syringes for injection with either CNO or Vehicle and noted the substance each animal was injected with. On the next experimental day, each animal would be injected with the other substance. The experimenter was unblinded after both experiments were completed.<br>For experiments involving Cre-negative littermates, the experimenter was blinded to the genotype until all behavioral assays were completed.

Reporting for specific materials, systems and methods

We require information from authors about some types of materials, experimental systems and methods used in many studies. Here, indicate whether each material, system or method listed is relevant to your study. If you are not sure if a list item applies to your research, read the appropriate section before selecting a response.

Materials & experimental systems

n/a	Involved in the study	
[ ]	[x]	Antibodies
[x]	[ ]	Eukaryotic cell lines
[x]	[ ]	Palaeontology and archaeology
[ ]	[x]	Animals and other organisms
[x]	[ ]	Clinical data
[x]	[ ]	Dual use research of concern
[x]	[ ]	Plants

Methods

n/a	Involved in the study	
[x]	[ ]	ChIP-seq
[x]	[ ]	Flow cytometry
[x]	[ ]	MRI-based neuroimaging

Antibodies

Antibodies used

Primary antibodies: Chicken anti-GFP (1:1000, LifeTech A10262), Goat anti-mCherry (1:500, Sicgen AB0081-200), Rabbit anti-mCherry (1:500, Thermo Fisher Scientific PA5-34974), Guinea pig anti-NeuN (1:1000, Synaptic systems 266004), Rabbit anti-c-Fos (1:500, Calbiochem PC38) Rabbit anti-c-Fos (1:100, Cell Signalling 2250), Rabbit anti-TPH2 (1:1000, Novus Biologicals NB100-74555), Goat anti-5-HT (1:500, Immunostar 20079), Rabbit anti-vGAT (1:500, Synaptic Systems 131003), Guinea pig anti-vGAT (1:500, Thermo Fisher Scientific PA5-111832), Alexa 488-Donkey anti-Chicken (1:500, Jackson ImmunoResearch 703-546-155), Cy3-Donkey anti-goat (1:500, Jackson ImmunoResearch 705-166-147), Alexa 488-Donkey anti-goat (1:500, Jackson ImmunoResearch 705-545-003), Cy5-Donkey anti-goat (1:500, Jackson ImmunoResearch 705-175-147), Alexa 488-Donkey anti-guinea pig (1:500, Jackson ImmunoResearch 706-545-148), Alexa 647-Donkey anti-guinea pig (1:500, Jackson ImmunoResearch 706-605-148), Cy5-Donkey antiguinea pig (1:500, Jackson ImmunoResearch 706-175-148), Alexa 488-Donkey anti-rabbit (1:500, Jackson ImmunoResearch 711-545-152), Alexa 647-Donkey anti-rabbit (1:500, Jackson ImmunoResearch 711-607-003), Cy3-Donkey anti-rabbit (1:500, Jackson ImmunoResearch 711-165-152)

Validation

All antibodies used have a research resource identifier (see key resources table), with links to manufacture guidelines and references validating the reliability of each antibody for detecting its antigen

Animals and other research organisms

Policy information about studies involving animals; ARRIVE guidelines recommended for reporting animal research, and Sex and Gender in Research

Laboratory animals

For behavioral and histological assays, animals >6weeks of age were used. For electrophysiological assays, animals >4weeks were used. Mice were housed at a maximum of 5 per cage and maintained on a 12-hr light/dark cycle in a temperature-controlled environment with ad libitum access to food and water. Transgenic animals or Cre-negative littermates were used for all experiments. All animals were purchased from Jackson Lab, vGATcre (#016962/ IMSR_JAX:016962 and #028862/IMSR_JAX:028862), TRAP2 (#030323/ IMSR_JAX:030323), Ai65 (#021875/ IMSR_JAX:021875)

Wild animals

Study did not involve wild animals

Reporting on sex

Both sexes were used for all experiments with the exception of fig 1A, fig. 3E,F, and extended data fig. 3 G-H

Field-collected samples

Study did not involve samples collected in the field

Ethics oversight

Experiments involving mice and performed at the University of Zurich were approved by, and conducted in accordance with, the Veterinaeramt des Kantons Zurich (licences, 154/2018 and 097/2021). Experiments using mice performed at the NIH/NIDCR followed NIH guidelines and were approved by the National Institute of Dental and Craniofacial Research ACUC.

Note that full information on the approval of the study protocol must also be provided in the manuscript.

Plants

Seed stocks

N/A

Novel plant genotypes

N/A

Authentication

N/A
